# The role of vitamin D in amelioration of oral lichen planus and its effect on salivary and tissue IFN-γ level: a randomized clinical trial

**DOI:** 10.1186/s12903-024-04239-0

**Published:** 2024-07-17

**Authors:** Rania Shalaby, Marwa El Nawawy, Khaled Selim, Samah Bahaa, Sahar El Refai, AbeerAbd El Maksoud, Mahitab El Sayed, Aya Essawy, Asmaa Elshaer, Mohamed ElShaer, Moataz Maher Kamel, Yasmine Gamil

**Affiliations:** 1 Oral Medicine, Diagnosis, and Periodontology, Faculty of Dentistry, Fayoum University, Fayoum, Egypt; 2 Oral Medicine, Diagnosis, and Periodontology, Faculty of Dentistry, Cairo University, Cairo, Egypt; 3 Oral Pathology, College of Dentistry, Princess Nourah Bint Abdulrahman University, Riyadh, Saudi Arabia; 4 Oral Biology, Faculty of Dentistry, Fayoum University, Fayoum, Egypt; 5 Clinical Pharmacy Department, Faculty of Pharmacy, Modern University for Technology and Information, Cairo, Egypt; 6 Clinical Pharmacology Department, Faculty of Medicine, Ain Shams University, Cairo, Egypt; 7 Medical Biochemistry and Molecular Biology, Faculty of Medicine, Cairo University, Giza, Egypt; 8 Department of Oral Medicine, Diagnosis, and Periodontology, Faculty of Oral and Dental Surgery, Modern University for Technology and Information, MTI University, Cairo, Egypt

**Keywords:** Oral lichen planus, Vitamin D, Oral potentially malignant lesions, Salivary IFN-γ, Vitamin D receptors, Interferon-gamma, OLP lesions

## Abstract

**Background and objectives:**

Oral lichen planus (OLP) is a common, prevalent, immune-mediated, inflammatory disease affecting both the skin and oral mucosa and is considered one of the potentially malignant diseases. Since OLP is regarded as an immunologically mediated disease, some studies suggest the use of vitamin D (VD) for its management as it exhibits immune-modulatory, anti-inflammatory, and antimicrobial properties, as well as anti-proliferative, pro-differentiative, and anti-angiogenic effects. VD has demonstrated a suppressive effect on TH1 pro-inflammatory cytokines, including IFN-γ while augmenting the secretion of anti-inflammatory cytokines. At the same time, VD deficiency is a prevalent public issue. Therefore, the present study aimed to investigate the role of VD as an adjunct to steroids in the management of VD-deficient OLP patients as well as its inhibitory effect on IFN-γ through measurement of salivary and tissue IFN-γ levels in OLP patients.

**Methods:**

A total of 40 patients with ulcerative or erythematous OLP, diagnosed according to the World Health Organization’s (WHO) modified criteria for OLP, were randomly allocated into one of the two study groups to receive either systemic steroids in addition to VD supplements (Group A) or systemic steroids only (Group B). Blood samples were collected for the measurement of serum VD level (SVDL) using the enzyme-linked immunosorbent assay (ELISA) to involve only patients with VD deficiency or insufficiency (≤ 30 ng/ml). Clinical evaluation of the lesion involved objective signs and subjective symptoms. Also, changes in salivary and tissue INF-γ levels (in pg/mL and pg/mg, respectively) were determined using the ELISA technique. All parameters were measured at baseline and after 4 weeks of treatment. The clinical pharmacy team devised a checklist to record all team interventions. The interventions were categorized into six domains, including drug interactions and/or adverse reactions, medication dose issues, drug selection issues, support with medication history, patient-related concerns, and suggestions for dental medication.

**Results:**

After one month of treatment, a significantly greater number of patients in group A showed complete pain relief and resolution of clinical lesions, as well as a greater number of patients showing a reduction in the clinical severity of lesions than in group B (*P* = 0.005). Also, there was a statistically significant reduction in average VAS pain scores and clinical scores in group A compared to group B after 1 month of treatment (*P* = 0.001 and 0.002, respectively). Furthermore, there was a statistically significant greater reduction in salivary and tissue IFN-γ levels in group A than in group B (*P* ≤ 0.001 and 0.029, respectively) after 1 month of treatment.

**Conclusion:**

Current evidence suggests a significant preventive and therapeutic role for VD as an adjunct to standard therapies indicated for OLP lesions. These protective and therapeutic functions are achieved through the suppressive effect of VD on pro-inflammatory cytokines, particularly IFN-γ. Also, salivary IFN-γ appears to be a valuable prognostic marker for monitoring the progression of OLP. In addition, the inter-professional collaboration between dentists and clinical pharmacists helped to deliver complete, patient-centered primary care and ensured the quality of the medications included in patient kits, thus improving patient treatment and management. Nevertheless, further studies with larger sample sizes, longer follow-ups, and standardized designs may still be needed.

## Introduction

Oral lichen planus (OLP) is a chronic inflammatory and immunologic disease involving mainly the oral mucosal tissues [1]. Generally, the prevalence of OLP in the public population ranges from 0.5 to 2.2%, affecting primarily females over 40 years of age [2]. Clinically, OLP presents in six different types: reticular, papular, or erythematous OLP, which are usually asymptomatic, while others, like erythematous (erythematous), ulcerative, or bullous OLP, are present with pain and/or a burning sensation [3, 4].

Despite substantial research, the etiopathogenesis of OLP remains ambiguous. However, dysregulation of cell-mediated immunity has been implicated in the disease’s pathogenesis. This process is mediated by various cytokines that promote auto-reactive cytotoxic (CD8) T-cells, inducing keratinocyte apoptosis [5, 6]. A wide range of cytokines are produced by T-helper (TH) (CD4) cells, including TH1 and TH2, as well as T-regulatory cells (Tregs). The signaling between keratinocytes and inflammatory cells is mediated by these cytokines, which hence play a critical role in keratinocyte apoptosis [7]. Interferon gamma (INF-γ), being the principle cytokine produced by TH1 and TH2 cells, has been involved in the immunopathogenesis of OLP as it contributes to keratinocyte apoptosis, which is triggered by cytotoxic CD8 T-cells [7, 8].

Owing to the obscure nature of OLP, there is no fixed or definitive treatment regimen recommended for its management yet. In part, asymptomatic forms of OLP may not require any treatment, while several treatment modalities have been advocated for symptomatic forms of OLP [9–13]. Principally, most treatment strategies aim to relieve pain and burning sensations as well as hasten the remission of erythematous, ulcerative, or bullous lesions. Despite the multiple strategies that have been employed for the treatment of OLP, the greatest body of evidence indicates that the use of corticosteroids is the most efficacious treatment modality [4, 13]. Nevertheless, both topical and systemic steroids possess significant adverse effects that limit their use over an extended period of time. This is especially problematic in the management of OLP, which is a chronic disease exhibiting periods of remission and exacerbation. In addition, not all patients respond favorably to steroids. Therefore, it is vital to use an additional treatment modality that could augment the action of steroids which shortens the treatment duration and thus, minimize the possible adverse effects of steroids with better treatment outcome [14].

The active forms of vitamin D (1,25-dihydroxyvitamin D or calcitriol) were among the suggested therapeutic agents in the management of OLP [15, 16]. Vitamin D, the secosteroid, exhibits anti-angiogenetic, anti-proliferative, and pro-differentiative activities. These biologic actions could be achieved through the coupling of vitamin D to its receptors (VDR), which are abundantly present on different cells, including immune cells [17–20].

Evidence from existing literature refers to the association of vitamin D deficiency or insufficiency with many autoimmune and inflammatory disorders [21–24]. On the other hand, studies indicated that an increased dietary intake of vitamin D diminished the risk of inflammatory disease [25, 26]. This anti-inflammatory action of vitamin D has been ascribed to its ability to regulate the immune response through modulation of macrophage function, induction of dentiritic cell maturation, and suppression of TH-1 and TH-2 pro-inflammatory pathways, particularly that it could inhibit the production of INF-γ in the epithelium. Furthermore, in several human cancers, vitamin D shows pro-apoptotic functions and inhibits invasion and angiogenesis [17, 25–28].

Given these multiple protective and regulatory effects of vitamin D, bearing in mind the malignant potential of OLP, the aim of this study is to evaluate the efficacy of vitamin D as an adjunct to systemic steroids in the management of vitamin D-deficient OLP patients. Furthermore, we investigated the assumption that vitamin D could inhibit INF-γ production through the assessment of salivary and tissue INF-γ before and after treatment with steroids alone or in conjunction with vitamin D. To the best of our knowledge, no previous studies have investigated the correlation between the clinical outcome of adding vitamin D to the standard OLP treatment and changes in INF-γ levels in tissue and saliva providing evidence on the possible mechanism by which vitamin D could ameliorate OLP lesions.

## Methods

### Trial design

This study is a randomized clinical trial (RCT) with a parallel design and an allocation ratio of 1:1. The study conforms with the Consolidated Standards of Reporting Trials guidelines (CONSORT guidelines) [29].

### Study setting and recruitment

Eligible subjects were recruited over a period of three months, from the first of July until the first of October. Participants were selected from the clinics of the Department of Oral Medicine, Diagnosis, and Periodontology at the Faculty of Dentistry, Fayoum, Cairo, and MTI Universities. **Study protocol has been registered in ClinicalTrial.gov on 12/01/2024, protocol ID**: NCT06204796.

### Eligibility criteria

#### Inclusion criteria

Middle-aged patients presenting with clinical and histopathological features of symptomatic (erythematous, ulcerative, or bullous) OLP [30], and having vitamin D deficiency or insufficiency (≤ 30 ng/ml) [31] were included in the present study.

#### Exclusion criteria

The exclusion criteria involved any other oral mucosal lesion, any suspected restoration-related reaction, or active periodontitis. Also, patients should not be receiving any topical or systemic medication that may affect SVDL or induce a lichenoid reaction. Based on the modified Cornell Medical Index questionnaire [34], any patient with systemic disease was excluded.

### Sample size calculation

Sample size was calculated based on the previous study by Razi et al. [32]. We considered the difference in clinical severity at week 4 of treatment between the two independent study groups using t-tests with a probability of type I error (α) = 0.05 and a power (1-β) of 0.9. The estimated sample size was found to be 36 participants (18 in each group). The sample was increased to 40 (20 in each group) to allow for dropout loss. Sample size calculations were performed by the G*Power 3.1.9.7 program.

### Intervention and study groups

A total of 40 participants were randomly and equally allocated into one of the two study groups to receive either systemic steroids in addition to vitamin D supplements (**Group A)** or systemic steroids only (**Group B).**

### Outcome measures

#### Primary outcome

Clinical evaluation of the lesion involves two components, including objective morphological signs and subjective symptoms that describe pain and burning sensations, measured at baseline and after 4 weeks of treatment. The Scoring system is depicted in Table 1.

**Table 1:** Scoring system for the severity of clinical condition

Clinical parameters	Sub-site score A	Severity score B
Scores	0 = no lesion 1 = evidence of lichen planus 2 = ≥ 50% of buccal mucosa, dorsum of tongue, floor of mouth, hard palate, soft palate or oropharynx affected	0 = keratosis only 1 = keratosis with mild erythema (≤ 3 mm from gingival margin) 2 = marked erythema (e.g. full thickness of gingivae, extensive with atrophy or edema on non-keratinized mucosa) 3 = ulceration present
Severity of clinical appearance = A*B (sub-site score* severity score)	Mild : 1–2, Moderate: 3–4 and Severe: 5–6	
VAS score or burning sensation and pain ranging from 0 to 10	Mild : 0–4, Moderate: 5–7 and Severe: 8–10	

##### Objective morphological findings (signs)

According to Silverman et al. [33] and Escudier et al. [34], the severity of the clinical appearance of the OLP lesion was determined as the product of the multiplication of sub-site score A and severity score B. Finally, if the score is 0, the lesion is considered cured.

##### Subjective findings (symptoms)

VAS score for pain or burning sensation ranging from 0 to 10.

#### Secondary outcome

Changes in salivary and tissue INF-γ levels (in pg/mL and pg/mg, respectively) at baseline and after 4 weeks of treatment (measured using the ELISA technique).

### Clinical assessment, lab investigations, and implementation

At the beginning of the study, all participants were examined for the existence of signs and symptoms suggestive of ulcerative or erythematous oral lichen planus before being confirmed histologically according to the World Health Organization’s (WHO) modified criteria for OLP [35].

Then, 5 ml of peripheral venous blood were withdrawn from each patient for the measurement of serum vitamin D level (SVDL) using the enzyme-linked immunosorbent assay (ELISA) to involve only patients with vitamin D deficiency or insufficiency (≤ 30 ng/ml). SVDL was measured again after 1 month of treatment. Also, saliva and tissue samples were collected at the beginning and after 1 month of treatment for evaluation of the INF-γ level in tissue and saliva samples using the ELISA technique.

### Salivary sample collection

According to Navazesh [36], the collection of whole unstimulated saliva (WUS) samples took place between the hours of 9 a.m. and 11 a.m. Prior to saliva collection, participants were instructed to abstain from consuming any food or beverages for about 90 min. The participants were provided with instructions to incline their heads in a forward position, thereafter performing the actions of swallowing and expelling saliva into a designated tube for a duration of five minutes. The samples underwent centrifugation at a force of 4000 x g, and the resulting liquid portion (supernatant) was then preserved at a temperature of -20 ºC until the time of examination.

### Measurement of serum vitamin D level (ng/ml)

For the measurement of vitamin D3, the ELISA technique (supplied by ORGENTEC Diagnostika GmbH, Germany) was used to process all samples simultaneously. This test uses a competitive ELISA-based method that was designed to quantitatively determine the concentration of 25-OH vitamin D3/D2 in human serum or plasma.

In the first stage, 25-OH vitamin D3 in the sample was separated from vitamin D-binding protein. Then, samples were transferred to the reaction microwells of the microtitre plate. These microwells are coated with 25-OH vitamin D antibodies, with which both the separated 25-OH vitamin D in samples and the 25-OH vitamin D tracer reagent compete for binding to form complexes. Following incubation, the unattached and unspecifically bound molecules were eliminated in the first washing step. Subsequently, the immobilized tracer-antibody complex was coupled to an enzyme conjugate. After incubation, the unbound enzyme conjugate was removed in a second washing step. During incubation, the addition of enzyme substrate produced a blue color. Then, an acid was added to stop the reaction from generating a yellow-colored product. When evaluated photometrically at 450 nm, the degree of the yellow hue is inversely proportional to the amount of vitamin D present in the sample. Vitamin D levels lower than ng/mL were considered vitamin D insufficiency [30].

### Collection and preparation of tissue specimens

Tissue biopsy samples were obtained from the corresponding individuals in both groups 1 h after the collection of WUS. The biopsy specimens were immersed in a 10% formalin solution for fixation and then underwent dehydration using xylene and ethanol. The specimens were subsequently fixed in paraffin blocks and sliced into sections with a thickness of 4 micrometers using a microtome. A section of each sample was sent for standard histopathological analysis, while another section was submitted for tissue homogenization. In brief, the tissue samples weighing about 200 mg were subjected to homogenization in 2 ml of cold phosphate saline (PBS) with a pH of 7.4 and a concentration of 0.01 M. Prior to homogenization, the samples were triturated in liquid nitrogen and a homogenizer was employed. The homogenate samples underwent centrifuging with a force of 6000 xg for 20 min at a temperature of 4 °C. Subsequently, the supernatant of the samples was preserved at a temperature of 80 °C until it was employed for the purpose of detecting INF-γ through the utilization of ELISA.

### Histopathological examination

**Fig. 1:**
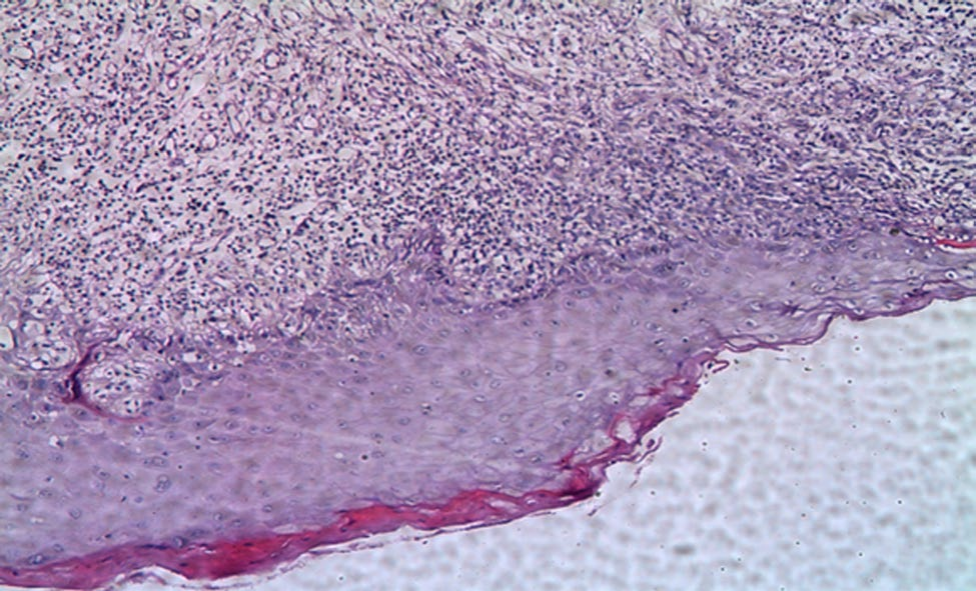
Photomicrograph of erythematous OLP showing thin epithelial thickness and a dense band of inflammatory cells infiltration, (H&E ×200)

The 4-µm-thickness sections were stained with hematoxylin and eosin (H&E) staining. Histopathologic examination was performed independently by two investigators (Oral pathologists) in a double-blind manner, utilizing the published criteria [35] to confirm that the pathological diagnosis was consistent with the clinical diagnosis as depicted in Fig. 1.

### Measurement of IFN-γ in saliva and lesional tissue samples

The concentration of IFN-γ the supernatant of tissue and WUS was determined using an ELISA (R&D Systems Inc., Minneapolis, MN, USA). This test applies the quantitative enzyme-based sandwich immunoassay method. IFN-γ has been pre-coated with an INF-γ-specific polyclonal antibody. Each of the 100 L standards and samples was pipetted into a distinct well, and the immobilized antibody bound any INF-γ present. After washing, 200 L of INF-γ-specific enzyme-attached polyclonal antibody were added to the wells. The antibody was introduced for a duration of 2 h. After washing the wells to eliminate any unattached antibody-enzyme reagent, 200 mL of a substrate solution was added. After 30 min, the quantity of INF-γ bound in the first stage was proportional to the amount of color that developed. The concentration of IFN-γ was calculated from the colorimetric optical density, which was read at 450 nm, and the amount of blue color was determined by the measurement of the degree of absorbance at 450 nm with a wavelength correction equivalent to 540–570 nm after the yellow color development ceased. Frequently, the minimal measurable dose (MDD) of INF-γ was ≤ 8.0 pg/mL. The measurement of total protein using the Protein Assay Kit (Thermo Scientific Pierce^™^ Micro BCA) and ELISA analysis were performed in duplicate, and the data were calculated from the means of two tests for each sample. Using the standard curve, the values of the curve ranged from 0 to 1000 pg/mL. The level of IFN-γ was presented as mg/ml in WUS and as pg/mg (the ratio of IFN-γ concentration to total protein) in tissues, as previously described [37].

### Treatment administration

All participants received a single morning dose of 40–60 mg of systemic prednisone according to the severity of the condition until the lesion size was reduced to 50%, then the dose was incrementally reduced by 10 mg each week to end up with 5 mg daily in the last week [38]. Patients were followed up weekly for up to 60 days. The length and dose of treatment were determined according to clinical requirements in each case. While group (B) received only systemic steroids, **group (A) was given** vitamin D supplementation at 60,000 IU weekly in conjunction with systemic steroids.

### Randomization and blinding

After patients’ consent to enrollment, an allocation sequence was performed using computer-generated randomization at a ratio of 1:1. The same calibrated investigator performed the clinical examination at baseline, and she was responsible for treatment implementation. The treatment couldn’t be blinded by the operator or patient. Another investigator examined patients after 1 month of treatment. Biomarker assessment and histopathologic examination were performed independently by two investigators (Oral pathologists) in a double-blind manner, and they were unaware of clinical examination and treatment delivery. The primary investigator, who was not involved in the assessment of outcomes or treatment implementation, received all data and performed statistics.

#### Data collection and follow-up

After the initial diagnosis visit (during recruitment), patients were clinically examined again for assessment of outcomes at baseline and after 1 month of treatment. Patients were followed up for up to 60 days.

### Interprofessional role of clinical pharmacists in dental clinics

The clinical pharmacy team devised a checklist for the purpose of recording all team interventions [39, 40]. The interventions were categorized into four domains, including drug adverse reactions, medication dose issues, patient-related concerns, and suggestions for dental medication.

The classification of such domains was determined after interaction with clinical pharmacists. The difficulties that were detected were appropriately directed to the relevant parties involved, namely the dentist and the patient. Furthermore, both the problem itself and the corresponding action were thoroughly recorded using a checklist. When a patient presented with any drug related problem, the clinical pharmacy team provided a comprehensive medication review and conducted screenings to identify potential opportunities for interventions, such as smoking cessation, lifestyle adjustments, or patient counseling. The aforementioned attempts were undertaken alongside dental professionals in order to facilitate collaboration and promote effective information sharing with the clinical pharmacy team.

## Statistcal methods

The normality of all quantitative variables was assessed using the Shapiro-Wilk test in order to choose suitable parametric or non-parametric tests. The mean and variance values were calculated for each group in every test. A paired sample t-test or a Wilcoxon signed rank test was utilized to evaluate the pre- and post-treatment measures within each group. In order to assess the differences in mean changes between the two groups for continuous data, either an independent sample t-test or a Mann-Whitney U test was utilized. The chi-square test, or Fischer’s exact test, was utilized to analyze binary data. Comparing the p-value to a predetermined threshold, frequently set at 0.05, allows for the determination of statistical significance. A Pearson’s correlation analysis was performed to investigate the hypothesized relationship between serum vitamin D levels and IFN-γ levels in tissues and saliva, with the latter being the dependent variable.

## Results

**Fig. 2:**
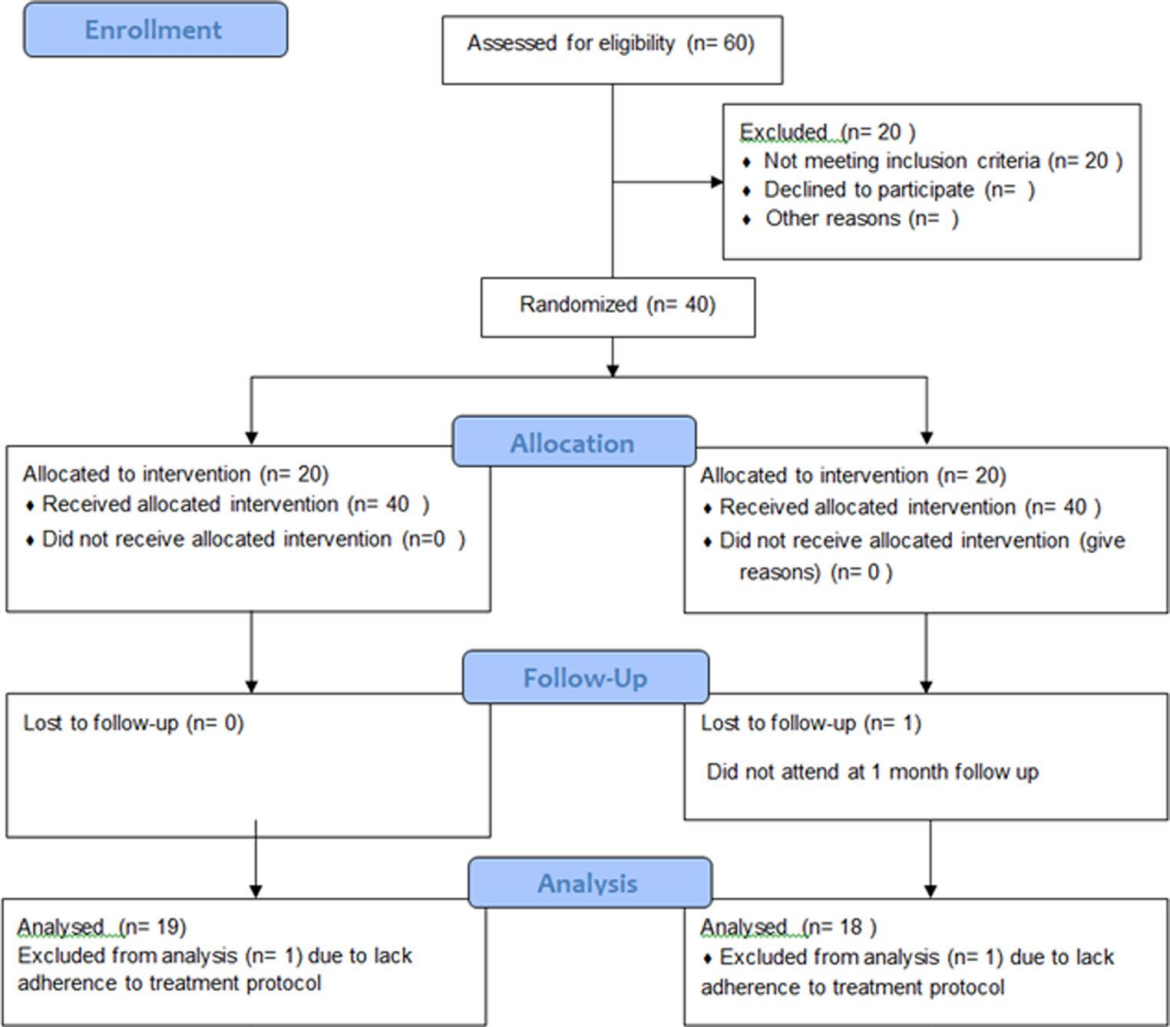
CONSORT flow diagram 2010

Out of 60 patients assessed for eligibility, 40 participants were randomized into the two study groups. In group A, 19 participants were included in the final analysis, and 18 participants from group B were included in the final analysis, as displayed in the flowchart (Fig. 2). The 37 participants consisted of 27 female patients, 14 from group A and 13 from group B, while there were 10 male patients, 5 from each group, with no statistically significant difference between the two groups. Also, there was no statistically significant difference in the mean of ages between the two groups, where the mean of ages of group A was 49.9 ± 8.87, whereas that of group B was 48.8 ± 9.08.

At baseline, there was no statistically significant difference between the two study groups in mean values (± SD) of any of the studied parameters, including pain (VAS) scores, clinical scores, or salivary and tissue IFN-levels (*P* = 0.902, 0.249, 0.843, and 0.766, respectively). Also, there was no significant difference in severity of pain or clinical picture between the two groups (*P* = 0.41 and 0.823, respectively), as shown in Table 1 and [2].

**Fig. 3:**
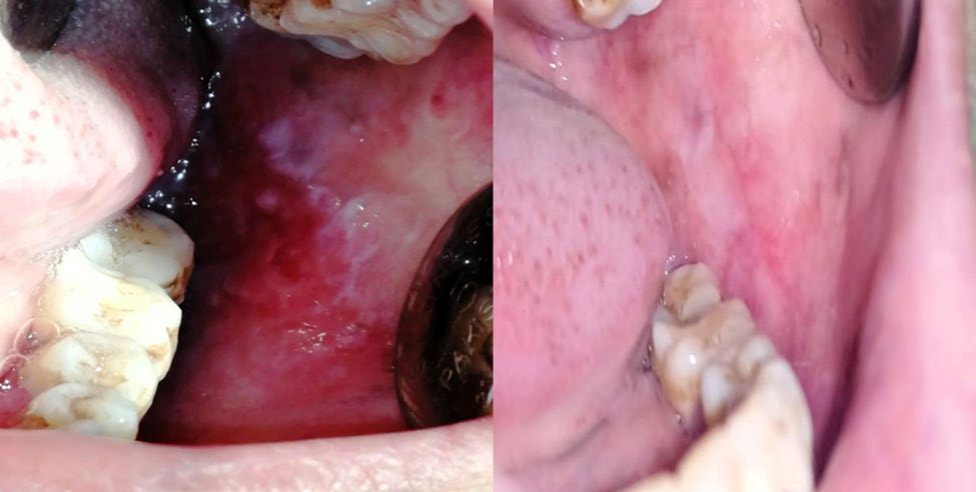
A clinical photograph of one of the ulcerative OLP lesions before and after treatment in group A

After one month of treatment, the majority of group A [10 (52.6%)] showed complete pain relief (cure) as compared to only 2 (11.1%) in group B, while 9 (47.4%) of group A shifted to mild pain versus 12 (66.7%) of group B. whereas none of group A showed moderate pain compared to 4 (22.2%) in group B, and no severe cases were found in any of the 2 groups. Thus, there was a statistically significant greater decrease in pain severity in group A than group B after 1 month of treatment (*P* = 0.005). Similarly, there was a statistically significant more reduction (*P* = 0.005) in severity of clinical picture in group A compared to group B after 1 month of treatment, in which 7 (36.8%) of the cases in group A showed complete clinical resolution versus only 2 (11.1%) in group B, and 11 (57.9%) of group A became moderate compared to 10 (55.6%) in group B, while only 1 (5.3%) in group A was moderate compared to 6 (33.3%) in group B. Furthermore, no clinically severe cases were detected in any of the 2 groups by 1 month of treatment, as shown in Table 2 and Fig. 3.

**Table 2:** Clinical severity at baseline and after 1 month of treatment

			Group A (*n* = 19)	Group B (*n* = 18)	*P*-value^F^
VAS pain scores	Baseline	Mild	12 (63.2)	8(44.4)	0.410
		Moderate	6(31.6)	7(38.9)	
		Severe	1(5.3)	3(16.7)	
	After 1 month	Cure	10(52.6)	2(11.1)	0.005*
		Mild	9(47.4)	12(66.7)	
		Moderate	0	4(22.2)	
		Severe	0	0	
Clinical scores	Baseline	Mild	0	0	0.823
		Moderate	7(36.8)	6(33.3)	
		Severe	12(63.2)	12(66.7)	
	After 1 month	Cure	7(36.8)	2(11.1)	0.05*
		Mild	11(57.9)	10(55.6)	
		Moderate	1(5.3)	6(33.3)	

**Fig. 4:**
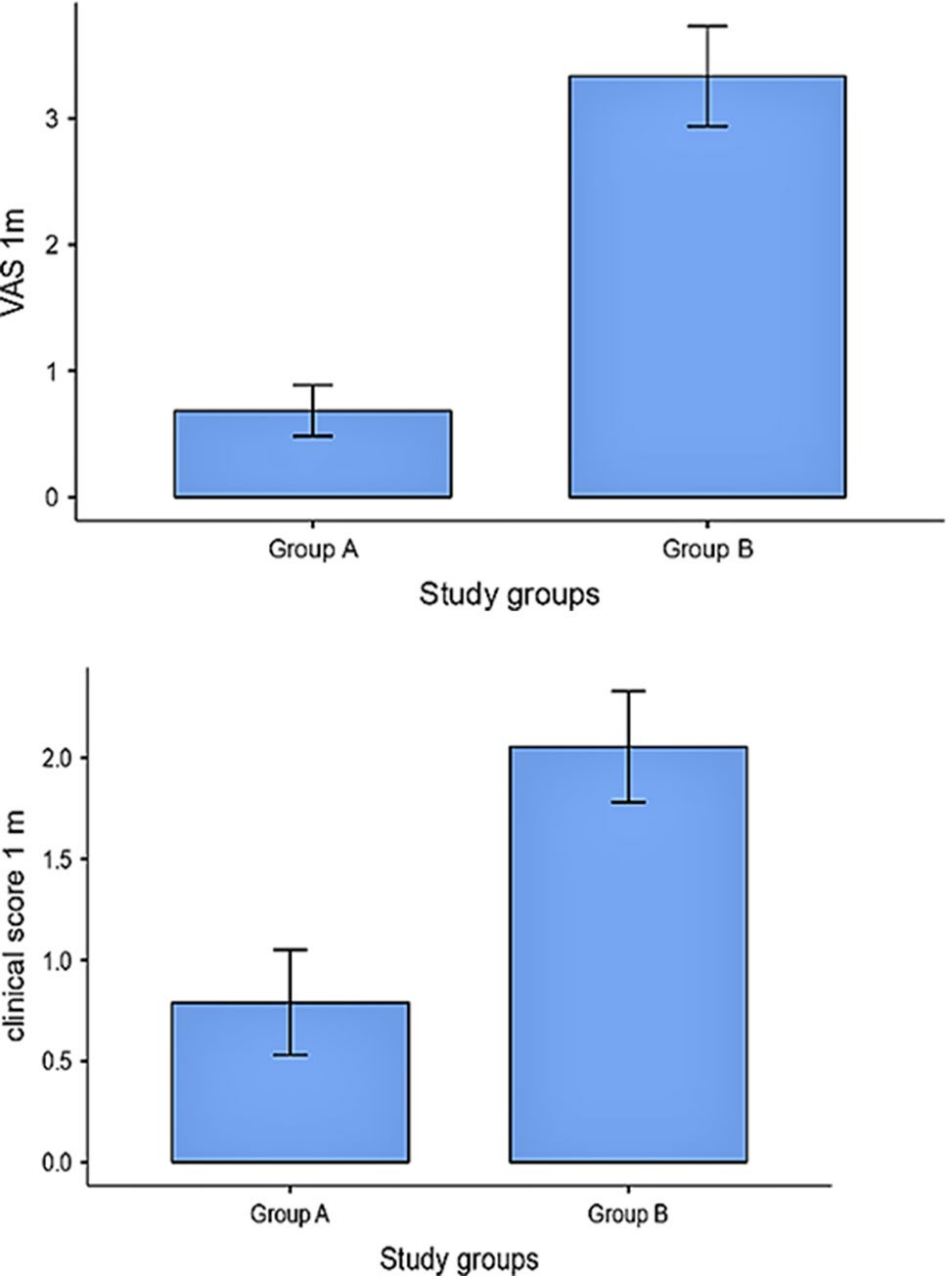
Difference in clinical severity after1 month between the two groups

**Fig. 5:**
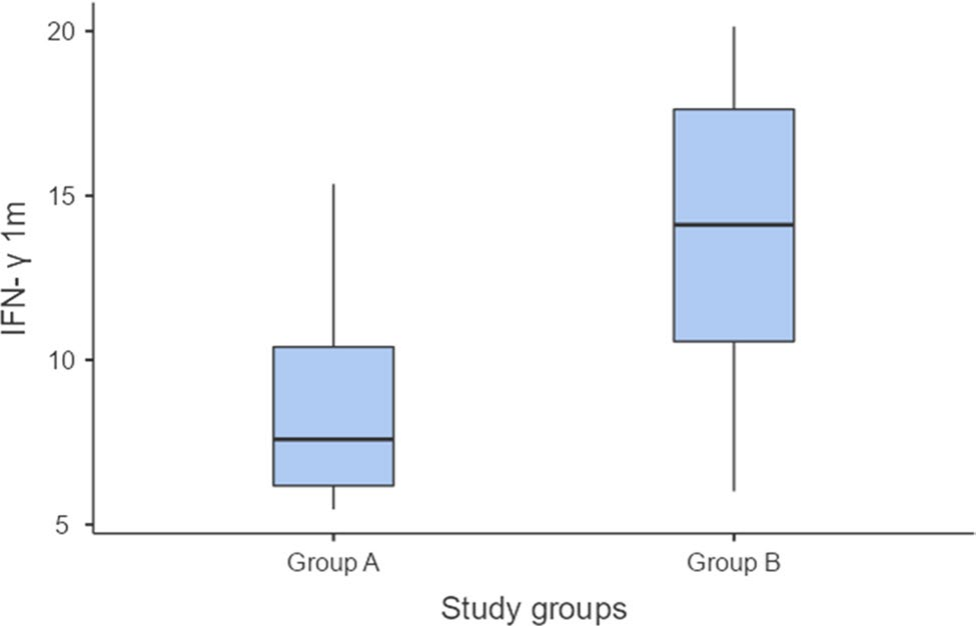
Difference in IFN-γ level after1 month between the two groups

**Fig. 6:**
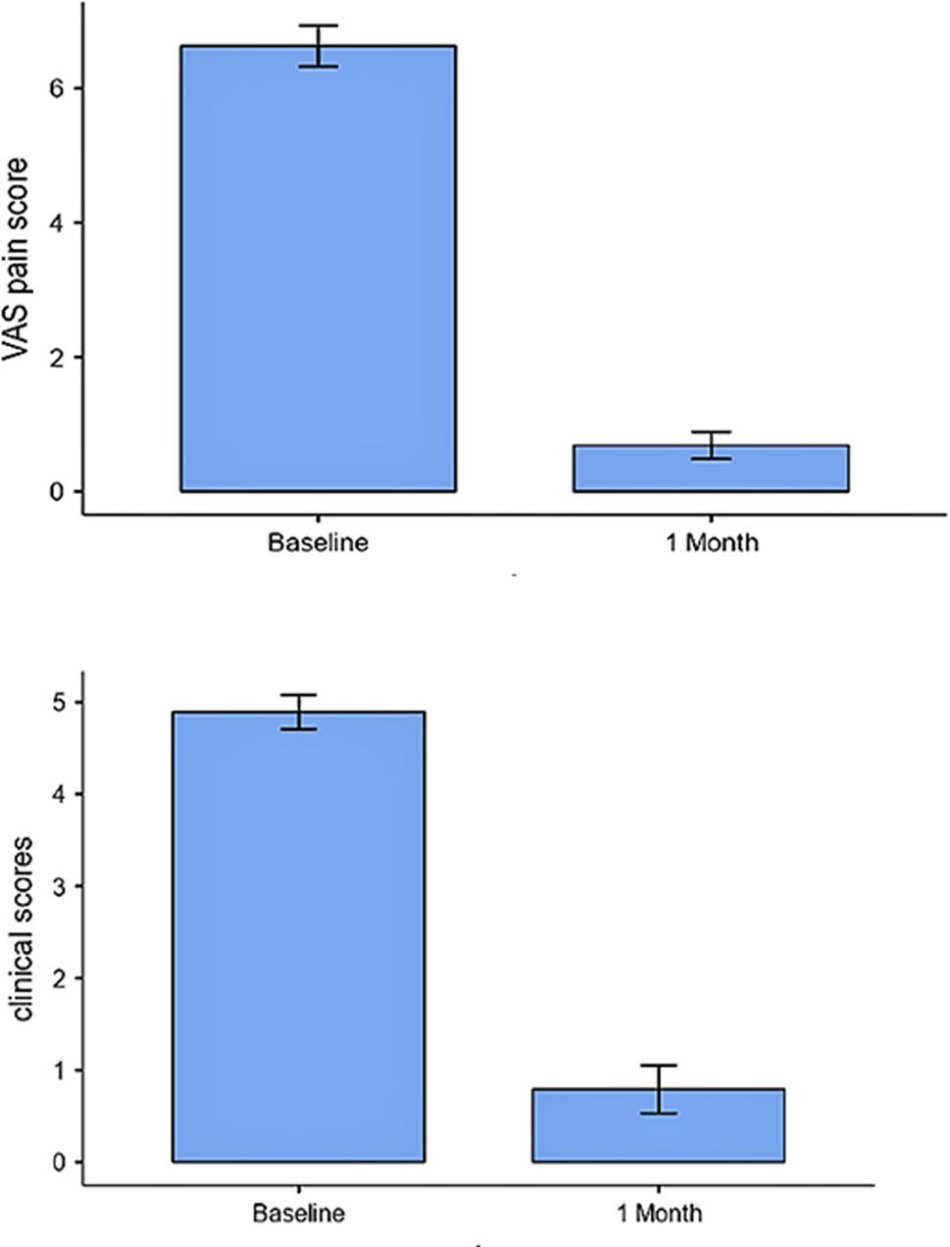
Difference in severity between baseline and after1 month in group A (vitamin D group)

**Fig. 7:**
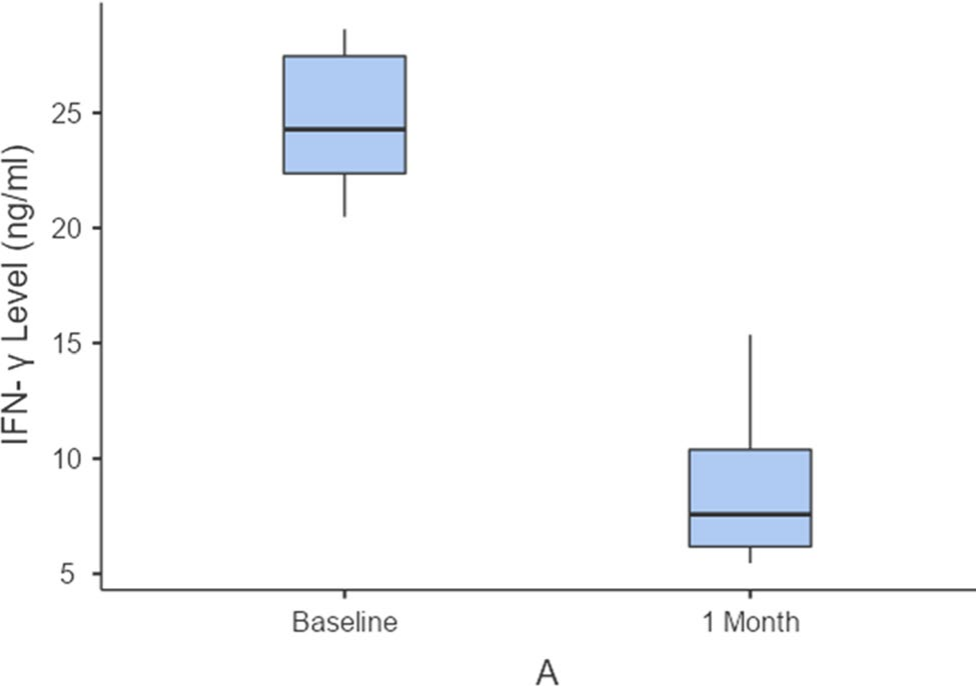
Difference in IFN-γ level between baseline and after1 month in group A (vitamin D group)

On the other hand, there was a statistically significant reduction in VAS pain scores and clinical scores in group A compared to group B after 1 month of treatment (*P* = 0.001 and 0.002, respectively) as displayed in Table 3 and Fig. 4. Likewise, there was a statistically significant greater reduction in salivary and tissue IFN-levels in group A than group B (*P* ≤ 0.001 and 0.029, respectively) after 1 month of treatment, as shown in Table 3 and Fig. 5. However, the difference between pre- and post-treatment values of pain and clinical scores as well as salivary and tissue IFN-levels was equally significant in the 2 groups (*P* ≤ 0.001), as shown in Table 4 and Figs. 6 and 7). Figure (3) shows one of the ulcerative OLP lesions before and after treatment in group A.

**Table 3:** Comparison between group A and group B regarding the studied variables

Parameters	Visit	Mean ± SD		Statistics	Mean difference	95% CI		Effect size	*P*-value
		Group A	Group B			lower	upper		
VAS pain score	At Baseline^a^	6.63 ± 1.34	6.67± 1.85	166.5	-5.93-6	-1.000	1.00	0.0263	0.902
	After 1 month^a^	0.684± 0.885	3.33 ± 1.68	35	-3.00	-4.00	-3.00	0.795	≤ 0.001*
Clinical scores	At Baseline^a^	4.89 ± 0.809	5.22 ± 0.943	135	-6.22-5	-1.000	5.8-6	0.2105	0.249
	After 1 month^a^	0.789 ± 1.134	2.06 ± 1.16	72	-1.000	-2.00	-1.00	0.576	0.002*
Salivary IFN-γ (pg/ml)	At Baseline^a^	24.66 ± 2.76	24.47 ± 2.62	164	0.211	-1.75	6.2	0.0409	0.843
	After 1 month^b^	8.56 ± 2.77	13.82 ± 4.62	-4.22	-5.26	-7.78	-2.73	-1.39	≤ 0.001*
Tissue IFN-γ (pg/mg)	At Baseline^b^	686 ± 124.7	673.40.2	0.3	13.1	-75.4	101.5	0.69	0.766
	After 1 month^b^	352 ± 64.3	40.2 ± 74.8	-2.82	-52.3	-98.7	-5.78	-0.75	0.029

**Table 4:** Comparison of the studied variables between baselines and after 1 month in the 2 study groups

Baseline − 1 month	Group	Test	Statistics	*P*-value	Mean difference	SE difference	95% CI		Effect size
							lower	upper	
VAS pain score	Group A	Wilcoxon W	190	≤ 0.001*	6.00	0.179	5.5	6.5	1.00
	Group B	Paired sample t’test	7.18	≤ 0.001*	3.33	0.464	2.35	4.31	1.69
Clinical scores	Group A	Wilcoxon W	190	≤ 0.001*	4.00	0.201	4.00	4.50	1.00
	Group B	Wilcoxon W	171	≤ 0.001*	3.00	0.218	2.5	3.5	1.00
IFN-γ (pg/ml)/	Group A	Paired sample t’test	17.9	≤ 0.001*	16.1	0.90	0.9	14.2	4.10
	Group B	Paired sample t’test	8.28	≤ 0.001*	10.65	1.287	7.94	13.37	1.95
Tissue IFN-γ (pg/mg)	Group A	Paired sample t’test	9.71	0.001	334	34.4	262	407	2.23
	Group B	Paired sample t’test							

Furthermore, results revealed a statistically significant correlation between changes in serum vitamin D levels before and after treatment in group A and salivary as well as tissue IFN- levels (*P* ≤ 0.001). Moreover, there was statistically significant correlation between changes in tissue IFN-γ, salivary IFN-γ as shown in Table 5.

**Table 5:** Correlation between tissue IFN-γ, salivary IFN-γ and serum vitamin D level

Correlation matrix		Salivary IFN level	Tissue IFN level
Serum vitamin D level	Pearson’s r	-0.906	-0.934
	*P*-value	≤ 0.001*	≤ 0.001*
Salivary IFN level (	Pearson’s r	-	0.919
	*P*-value	-	≤ 0.001*

## Discusssion

According to the World Health Organization (WHO), oral lichen planus (OLP) could be regarded as a common chronic autoimmune, inflammatory disease affecting primarily the skin and oral mucosa of unclear etiopathogeneis that has the potential for malignant transformation [39].

Despite its obscure etiology, it is generally considered an immune-mediated disease involving cytotoxic (CD8) T-lymphocytes that trigger keratinocyte apoptosis. Upon antigen presentation of the yet unidentified antigen by Langerhans cells, stimulation of helper (CD4) T-lymphocytes leads to up-regulation of T-helper 1 (TH1) cytokines and intercellular adhesion molecule-1 (ICAM-1), which further induce CD8 T-cell-mediated cytotoxicity against basal keratinocytes, causing basal cell degeneration [25, 41].

Although several treatment modalities have been advised for the management of OLP, corticosteroids remain the mainstay of its treatment. However, several drawbacks have been associated with the use of steroids in both topical and systemic preparations. Additionally, a variety of conditions are contraindicated for steroids, such as pregnancy and lactation, hypertension, diabetes mellitus, tuberculosis and herpetic infections, glaucoma, and human immunodeficiency virus (HIV) infection. Given the persistent and protracted course of OLP, together with the several adverse effects of steroids, it appears essential to find alternative treatment strategies for the management of OLP [42].

In the context of the immune process, vitamin D exhibits immune-modulatory, anti-inflammatory, and antimicrobial properties, as well as anti-proliferative, pro-differentiative, and anti-angiogenic effects. The active metabolite of vitamin D, calcitriol, exerts its action through binding to vitamin D receptors (VDR) present on multiple cells, including immune cells [17].

Vitamin D has immune regulatory effects by inhibiting the production of TH1 pro-inflammatory cytokines (e.g., interleukin-6 (IL-6), IL-17, IL-1b, IFN-γ, and TNF-α) while enhancing the secretion of anti-inflammatory cytokines (e.g., IL-4, IL-5, and IL-10). In addition, vitamin D and its active component (calcitriol) are believed to play a key role in a number of autoimmune diseases [21, 42]. This is achieved through inhibition of B-lymphocyte differentiation and proliferation, thus reducing the production of antibodies associated with autoimmune disorders [17, 43].

Meanwhile, vitamin D deficiency is an intimidating public health problem. Worldwide, the prevalence of vitamin D insufficiency was approximately 15.7% between 2000 and 2022 [44]. Generally, increased vulnerability to vitamin D has been related to increased indoor activities, especially during the COVID-19 pandemic. Moreover, populations with increased melanin pigmentation (due to decreased vitamin D synthesis even with adequate exposure to sunlight) are more susceptible to vitamin D deficiency. In particular, middle-eastern countries, including Egypt, show an increased prevalence of vitamin D deficiency. This may be attributable to some social, cultural, and religious practices that involve skin over-protection with extensive clothes or excessive use of sunscreens, as well as the limitation of certain types of food that are rich in vitamin D [4].

Considering the seriousness of vitamin D deficiency as a public health concern as well as the high prevalence of OLP and the need for an alternative to steroids in its management, together with the immune-modulatory effect of vitamin D that involves down-regulation of pro-inflammatory cytokines such as IFN-γ, we conducted the present study to investigate the role of vitamin D in the management of vitamin D-deficient OLP patients. In addition, we aimed to verify the suppressive effect of vitamin D on IFN-γ in OLP saliva and tissue samples, as it is the primary cytokine produced by TH1 cells.

In the present study, only cases confirmed by both clinical and histopathologic criteria based on the WHO modified criteria for OLP were included. Although in some OLP lesions the typical bilaterally symmetrical distribution of reticular, erythematous, or bullous ulcerative lesions associated with interlacing Whickham’s striae and burning sensations is usually diagnostic [38, 44], the histopathologic examination is imperative not only to validate the diagnosis but also to detect any signs of dysplasia [4].

Existing published literature emphasizes the need for a universal, comprehensive scoring system for clinical evaluation of OLP to standardize measurements of treatment outcomes. In previous clinical trials performed on OLP patients, several scoring systems have been employed, such as those of Thongprasom et al. [45], Piboonniyom S-O et al. [46], Elsabagh et al. [47], Chainani-Wu N et al. [48], Kaliakatsou F et al. [49], Escudier M et al. [34], and a combination of Silverman et al. [33] and Escudier et al. [34]. In accordance with Razi et al. [32], our study adopted the same scoring systems for the assessment of clinical outcomes. Furthermore, in this study, only vitamin D-deficient or insufficient (≤ 30 ng/ml) OLP patients were included to avoid the risk of vitamin D toxicity. This was considered based on the US Endocrine Society and International Osteoporosis Foundation guidelines, which regarded vitamin D levels of 21–29 ng/ml as insufficient [50].

The results of the present study revealed significantly greater improvement in both subjective and objective clinical outcomes in the group that received vitamin D in addition to systemic steroids than in the group that received systemic steroids alone. Additionally, greater number of patients showing complete clinical resolution were found in the group that received vitamin D than in the other group.

In a recent systematic review by Saeed et al. [4] that involved five studies, two from India ([51, 52] and one from each of Egypt [53]), Pakistan [32], and Iran [54] with a total of 714 participants, it was concluded that the use of vitamin D supplements in conjunction with conventional steroid therapy could significantly improve the signs and symptoms of OLP. Nevertheless, their results should be interpreted cautiously as they reported significant heterogeneity between the included studies involving variations in study design, intervention, number of participants, eligibility criteria, and the measured outcomes, which did not allow for meta-analysis and confers some doubt on a definitive conclusion.

The results of the current study were in line with three clinical trials [32, 53, 54] that compared the impact of vitamin D supplementation to steroid therapy in terms of changes in the clinical appearance of the OLP lesions and pain scores using the VAS scale. Our results were in accordance with Razi et al. [32] who used the same clinical scoring system as the present study, as they noticed significant amelioration in the clinical picture of OLP between 1 and 4 weeks of receiving vitamin D supplements combined with conventional therapy. Similarly, in a study from Egypt by Shoukeba et al. [53] a statistically significant reduction in VAS pain scores in both vitamin D and steroid-receiving groups was observed. In addition, their results revealed a 100% reduction in the size of the lesion after 6 weeks of treatment. However, in these two studies, the diagnosis of OLP relied on the characteristic clinical features without histologic confirmation.

Consistently, the results of Delavarian et al. [54] revealed a significant decrease in the severity of OLP lesions in the vitamin D, steroids, and lactose groups compared to the control group. It is worth mentioning that their results were based on WHO clinical and histopathologically modified criteria for the diagnosis of OLP, as in the current study.

Taking into consideration the psychological factor in the etiopathogenesis of OLP, two observational studies from India introduced psychological counseling to vitamin D and steroid therapy [51, 52]. They reported significant improvement in symptoms in OLP patients who received vitamin D. In particular, those who received both vitamin D and psychological counseling showed a marked diminution in pain and burning sensation. However, their diagnosis of OLP was primarily based on clinical features, whereas they restricted histopathologic examination to doubtful cases.

In the current study, the secondary outcome was the effect of vitamin D supplementation in conjunction with systemic steroids on salivary IFN-γ levels. IFN-γ is a major TH1 and TH2 cytokine that modulates the differentiation of T-cells, activates the maturation of cytotoxic (CD8) T-cells, and controls the integrity of MHC-II. Therefore, IFN-γ plays a significant role in keratinocyte apoptosis caused by CD8 + T cells in OLP [3].

Of interest, it has been evident from previous studies that IFN-γ concentrations in saliva were significantly correlated with their levels in lesion and serum samples. Thus, saliva can be an easy, effective, and non-invasive alternative to blood samples for the detection of IFN-γ levels in OLP patients. Notably, it is still controversial whether there is an increase or decrease in salivary IFN-γ levels in OLP patients compared to controls.

A systematic review [3] conducted a meta-analysis on the results of 11 studies involving various countries and a total of 442 OLP patients and 300 controls. The meta-analysis revealed that the levels with IFN-γ in the serum and saliva of OLP patients were comparable to those of healthy controls but substantially lower in ulcerative forms of OLP than in non-ulcerative forms. In contrast, their sensitivity analyses revealed significantly lower serum levels of IFN-γ in OLP patients compared to controls, as well as significantly lower salivary IFN-γ levels in ulcerative types compared to non-ulcerative types.

In four studies [25, 55–57], salivary IFN-γ was significantly higher in OLP patients than controls, particularly in erythematous and ulcerative forms of OLP. These findings signify the possible association between elevated IFN-γ levels and the severity of OLP lesions. Conversely, in three studies [58–60], lower levels of IFN-γ were detected in the saliva samples of OLP patients than those of controls.

In the present study, there were significantly lower levels of both salivary and tissue IFN-γ in the group that received vitamin D in addition to systemic steroids than in the other group, which received only systemic steroids after 1 month of therapy. However, there was a statistically significant difference between pre- and post-treatment values in the two study groups. These results could generally indicate that salivary and tissue IFN-γ levels were lowered with systemic steroid treatment, whereas a more pronounced reduction occurred with vitamin D treatment. Based on existing literature, the results of the present study are consistent with a study by Ghallab et al. [55] who found a statistically significant reduction in salivary IFN-γ levels after treatment with systemic steroids. It is noteworthy that saliva is considered a successful, simple, and non-invasive diagnostic tool that could serve in the detection of pro-inflammatory cytokines in several immunologic diseases [61].

In a cultured cell model [25], vitamin D blocked the lipopolysaccharide (LPS)-induced up-regulation of the nuclear factor kappa beta (NF-κβ) pathway that down-regulated the LPS-induced overproduction of pro-inflammatory cytokines in keratinocyte cells, implying that vitamin D supplementation may be a potential strategy for OLP management. Similar results were obtained from two other studies [62–64], highlighting the impact of vitamin D on the regulation of the NF-κβ pathway, thus reducing the production of IFN-γ.

In a recent study that was performed on COVID-19 patients [65], they found a significant negative correlation between vitamin D levels and IFN-γ levels (*P* = 0.0006), which could support the notion that vitamin D could suppress the inflammatory process through inhibition of IFN-γ production.

Consistently, in a recent study by Maboshe et al. [66] that used 400 IU of vitamin D3 supplement to manage seasonal vitamin D deficiency, they found that vitamin D attenuated the seasonal elevation of IFN-γ by about 28%, suggesting that vitamin D caused a diminution in the effector response associated with inflammation. Other studies [67, 68] discussed the protective function of vitamin D and VDR, unveiling their role in regulating microRNA (mi RNA) 27 a/b and mi RNA 26 a/b and thus inhibiting apoptosis and reducing pro-inflammatory cytokines, including IFN-γ, in epithelial cells.

An additional aspect worth considering is the chronic nature of OLP, as chronic diseases frequently necessitate the use of multiple medications (polypharmacy), which increases the risk of drug interactions and adverse effects, patient confusion, and noncompliance with the prescribed medications. Interprofessional care teams that include pharmacists can provide strategies to improve clinical outcomes, minimize side effects, and lower treatment costs by promoting patient therapeutic adherence. It was evident from the literature that patients with chronic diseases who obtain care from multidisciplinary teams of professionals have experienced better therapeutic results and higher levels of service satisfaction than those who receive care from one profession [40]. In this study, the cooperation between dentists and clinical pharmacists resulted in improvements in patients’ treatment and management. Inter-professional healthcare teams have delivered complete, patient-centered primary care that is population-based and ensured the quality of the medications included in patient kits.

## Conclusion

Overall evidence from current and previous studies suggests a significant preventive and therapeutic role for vitamin D as an adjunct to standard therapies indicated for OLP lesions. These protective and therapeutic functions are achieved through the suppressive effect of vitamin D on pro-inflammatory cytokines, particularly IFN-γ. Also, the present study corroborates the suggested prognostic value of salivary IFN-γ in monitoring the progression of OLP.

### Limitations

The present study’s constraints include a limited sample size and the absence of long-term follow-up. Furthermore, it has been assumed that liquid chromatography/tandem mass spectrometry (LC-MS/MS) is the ideal method for vitamin D analysis [19]; nevertheless, the ELISA technique is the method used in the present study. Moreover, the scarcity of studies with standardized designs did not allow adequate comparison to reach a final conclusion. Therefore, further studies with larger sample sizes, longer follow-ups, and standardized designs may be needed to reach a definitive conclusion.

## Data Availability

All data generated or analyzed during this study are included in this published article.
